# How well is messaging about the importance of vaccination for people living with dementia being communicated? A jurisdictional scan of National Immunization Technical Advisory Groups and dementia advocacy organizations

**DOI:** 10.1002/alz70860_097858

**Published:** 2025-12-23

**Authors:** Melissa K. Andrew, Shanna Claire Trenaman

**Affiliations:** ^1^ Dalhousie University, Halifax, NS, Canada

## Abstract

**Background:**

Vaccination is particularly important for people living with dementia (PLWD). Even so, vaccine uptake remains suboptimal, and messaging may not be optimally tailored. Awareness of PLWD as an important population for immunization may also be limited in public health settings, including in the work of National Immunization Technical Advisory Groups (NITAGs).

This study investigates: 1) Geriatrics representation in NITAG membership, 2) Whether dementia is considered a high‐risk condition in NITAG guidance, and 3) Whether national dementia advocacy organizations (e.g. Alzheimer Associations/Societies) specifically recommend vaccination as part of messaging on living well with dementia.

**Method:**

A jurisdictional scan was conducted among NITAGs and national dementia advocacy organizations in the following countries: Canada, USA, United Kingdom, Australia, Germany, France, Switzerland, and for the World Health Organization. Lists of each NITAG's voting members were reviewed, including institutional affiliations/biographies. Published recommendations were reviewed for five adult vaccines (COVID‐19, influenza, pneumococcal, RSV and Herpes Zoster) to determine whether dementia or related conditions appeared on lists of people for whom vaccination is particularly recommended. Websites for dementia advocacy organizations in each country were searched for information relating to vaccines, and any specific recommendation on the importance of vaccination for PLWD.

**Result:**

Four of the eight NITAGs (Canada, USA, France, Germany) included a geriatrician voting member. All jurisdictions had variations on age‐based recommendations for adult vaccines (e.g. recommending everyone over a certain age should be vaccinated). Some included specific mention of dementia as a high‐risk condition (eg UK, Germany, and France for COVID, Canada for RSV), and several included chronic neurological conditions (sometimes with Central Nervous System or neurodegenerative as descriptors). All but one of the dementia advocacy organizations made some mention of COVID vaccines. Only the USA and UK organizations mentioned any non‐COVID vaccine. None discussed vaccination as a specific recommendation for living well with dementia.

**Conclusion:**

Although geriatrics representation is increasingly part of NITAGs, and PLWD are sometimes included among high‐risk groups specifically recommended to receive vaccination, translation into public health and advocacy organization messaging remains suboptimal. This represents a missed opportunity and area for improvement given the benefits of vaccination for PLWD.

